# Supplementary figures and images for: Molecular basis of C9orf72 poly-PR interference with the β-karyopherin family of nuclear transport receptors
Source: Sci Rep. 2022 Dec 9;12:21324. doi: 10.1038/s41598-022-25732-y (PMC9734553; doi:10.1038/s41598-022-25732-y)

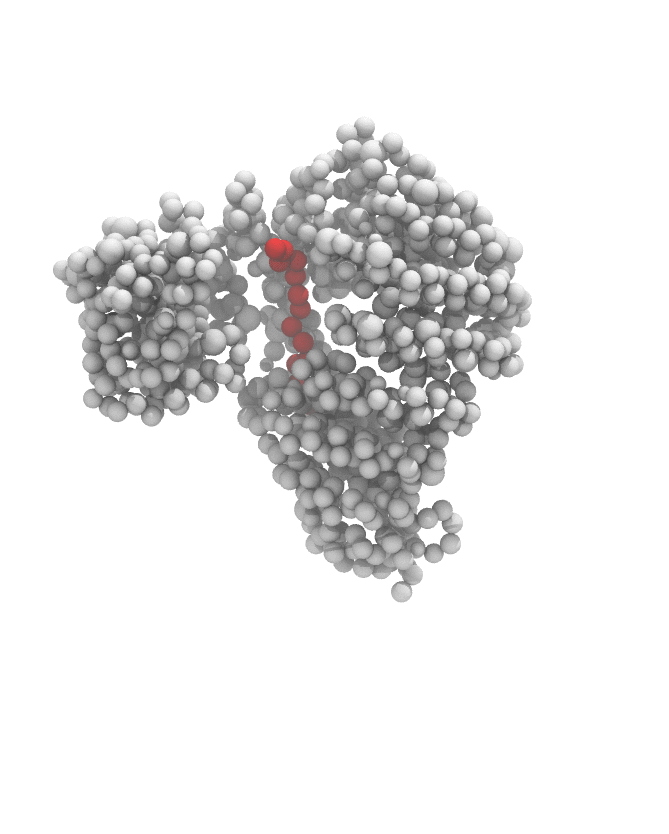

Supplement: Supplementary file 2 — Supplementary Movie S1. [file 41598_2022_25732_MOESM2_ESM.gif]

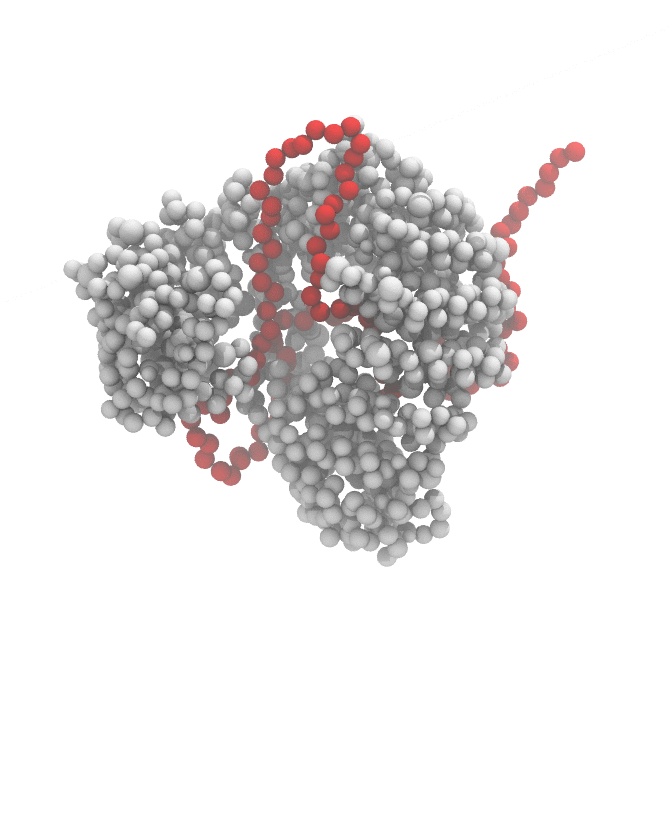

Supplement: Supplementary file 3 — Supplementary Movie S2. [file 41598_2022_25732_MOESM3_ESM.gif]

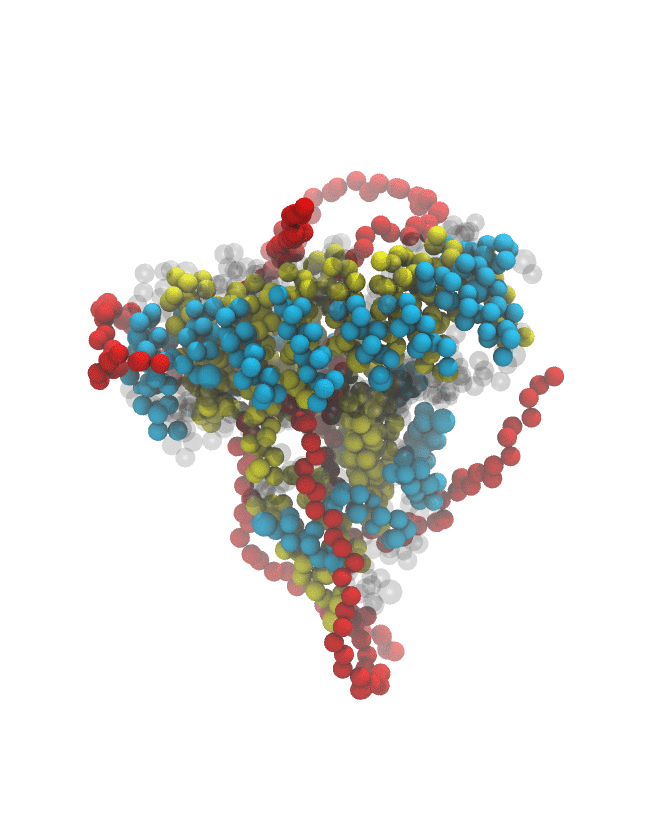

Supplement: Supplementary file 4 — Supplementary Movie S3. [file 41598_2022_25732_MOESM4_ESM.gif]
